# A Rare Case of Mesenteric Chylous Cyst in Infant: Case Report and Review of Literature

**DOI:** 10.3389/fsurg.2021.666488

**Published:** 2021-06-14

**Authors:** Alessia Salatto, Flavia Indrio, Vittoria Campanella, Marina Curci, Cosetta Maggipinto, Raffaella Cocomazzi, Francesco Canale, Maria Nobili, Fabio Bartoli

**Affiliations:** Department of Medical and Surgical Science, University of Foggia, Foggia, Italy

**Keywords:** chylous, mesenteric cyst, lymphatic malformation, laparacospy, laparotomy

## Abstract

The occurrence of a mesenteric cyst (MC) is common in adults while in children and in infants is rare. In adults mesenteric cysts are often asymptomatic and discovered incidentally; however, in children they commonly present with symptoms of abdominal pain or distension with fever and leucocytosis. We report on a rare case, in our experience, of Mesenteric Chylous cyst (MCC) in an infant with signs and symptoms of intestinal obstruction. Discussion of literature is also reported.

## Introduction

Mesenteric cyst (MC) is common in adults while in children and in infants is extremely rare (1–3). Clinical presentation may range from asymptomatic, a lump or pain in abdomen to intestinal obstruction or hemorrhage (4, 5). Mesenteric cyst in children, in general, are rare and benign lesions and often asymptomatic, but there are a several complications, including “dramatic” ones, related to these malformations: volvulus, necrotic and/or perforated bowel, infections. Differential diagnosis should be with lymphangioma, intestinal duplication or retroperitoneal cyst (6). Despite ultrasound may provide useful information, diagnosis is often made during surgical intervention and confirmed by pathologist. Here is an observational and clinical study about “mesenteric chylous cyst” in children, to evaluate the relationship between the “presence” of a MCC and problems related to any surgery.

## Case Report

**Figure 1:**
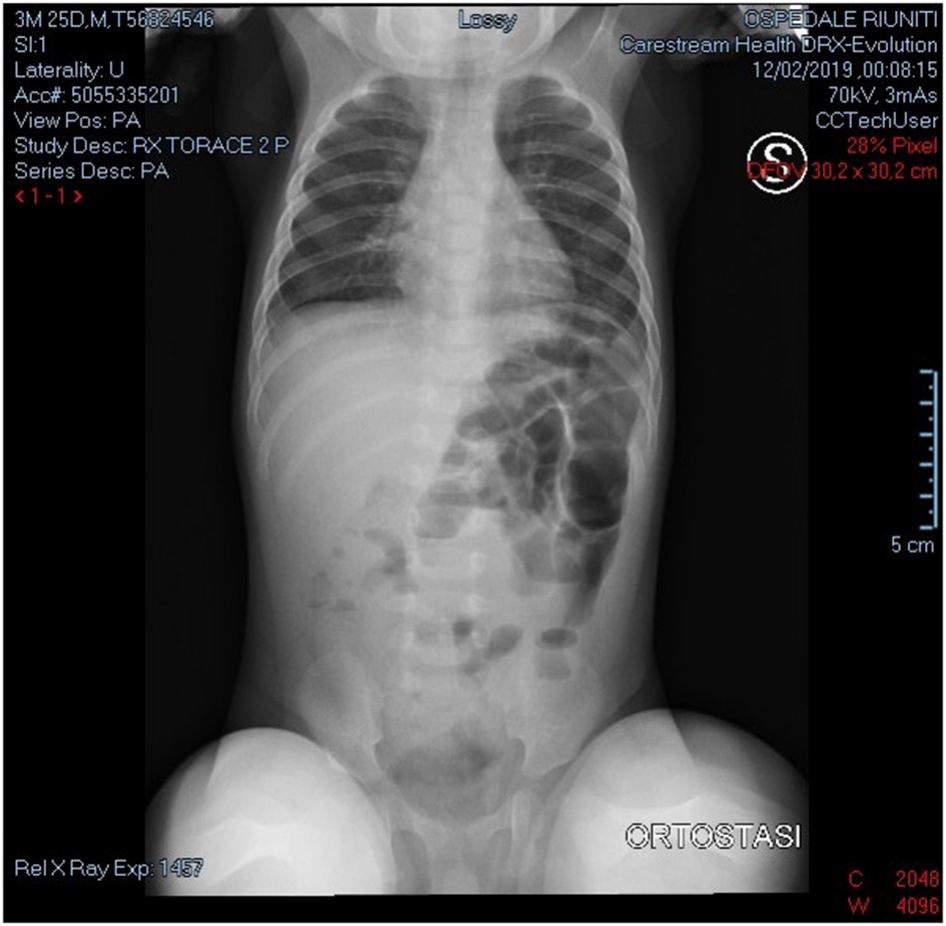
Plain abdominal x-ray showed air fluid levels.

**Figure 2:**
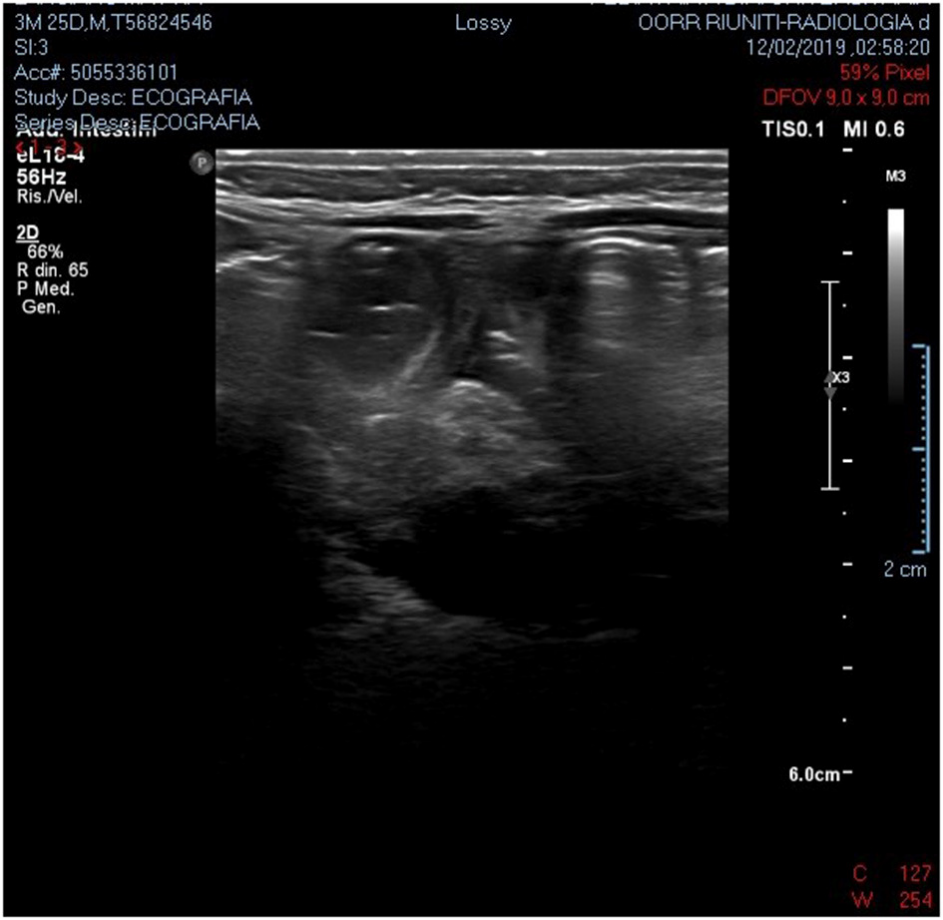
Abdominal US demonstrated a large sub hepatic fluid collection.

**Figure 3:**
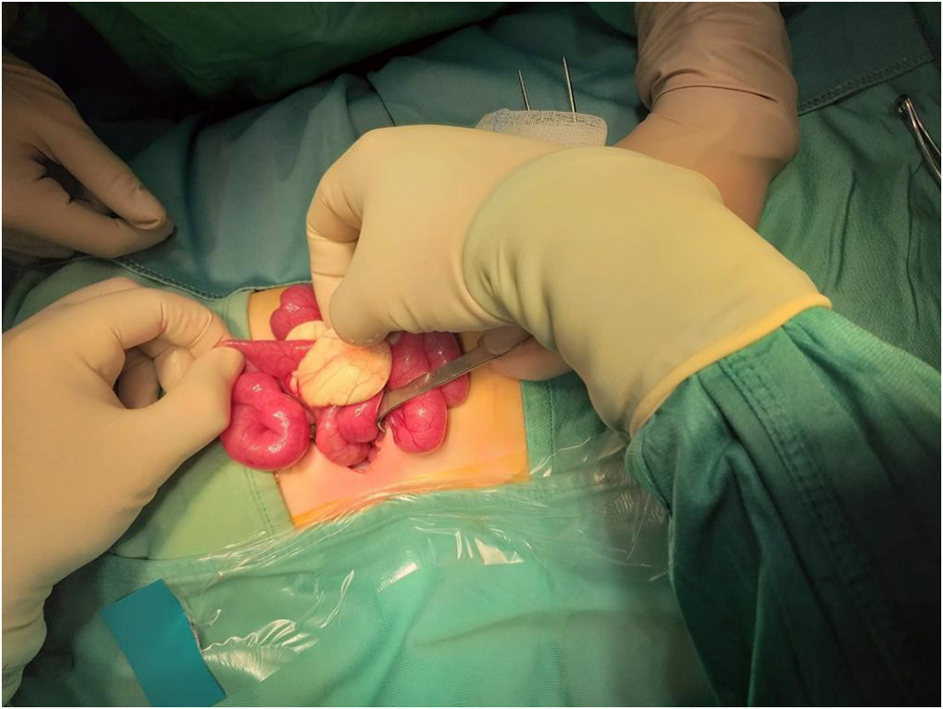
Laparotomy confirmed the presence of mesenteric cyst in the first jejunal loop, 7 cm in diameter.

**Figure 4:**
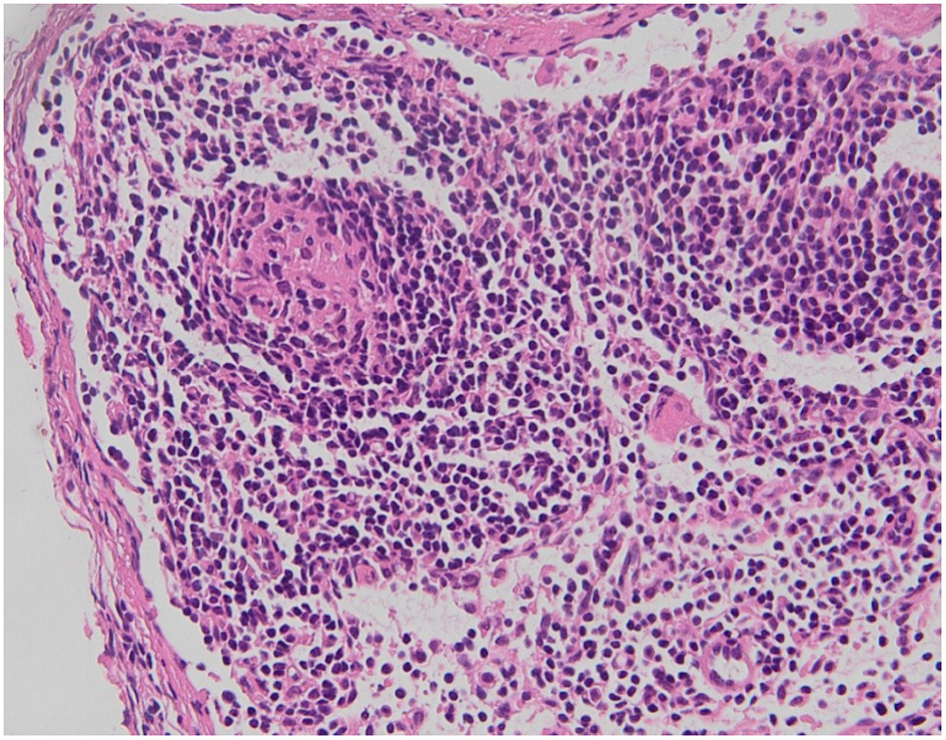
Histology of mesenteric leaves interested by intense chronic inflammation.

We present a case of a baby boy, born at 37 weeks GA (gestational age). The prenatal ultrasound performed in the third trimester of pregnancy showed any anomalies. At birth he had no clinical signs of obstruction. At 4 months of age he was hospitalized for bloody stool, fever and bilious vomiting. He was febrile to 38.9 °C and tachycardic to 205 beats per minute, with a blood pressure of 88/45. On physical exploration the abdomen was soft and mildly distended. White blood cell count and PCR were severely increased. Plain abdominal x-ray showed air fluid levels (Figure 1). Abdominal US demonstrated only a large sub hepatic fluid collection (Figure 2). The child underwent to explorative laparoscopy as diagnostic procedure to exclude intestinal obstruction or intestinal volvulus. The exploration revealed an extended inflammation of jejunal loops with large and white mesenteric cyst. Immediately, laparoscopy was converted in mini-laparotomy which confirmed the presence of MC in the first jejunal loop, 7 cm in diameter (Figure 3). Furthermore, we observed several reactive lymph nodes with a widespread dissemination of very small white lesions in the contest of all mesentery. An attempt was made to dissect the cyst but, as soon as we incised the mesentery, the cyst completely empty filling with liquid the mesenteric leaves. The jejunal loop wall also affected by necrosis and inflammation so we decided to resect a limited segment of it (3 cm), followed by jejunal end to end anastomosis and appendectomy. The specimens analyzed by pathologist, described the cyst as unilocular without a pseudo wall and confirmed that mesenteric leaves were interested by intense chronic inflammation without a lining epithelium (Figure 4). The muscular layer of resected bowel segment was interested by congestion and hemorrhage.

The infant reached the full enteral feeding on day 3rd post-operative and discharged home in good condition at day 6th. The follow up performed for 1, 2, and 4 years after surgery did not showed recurrence.

## Discussion

We report a baby boy 4 month old with chylous MC. The chylous mesenteric cyst is rarely been reported in the literature. Those cysts are commonly observed in adults but rarely seen in children (6). MC are considered as benign intra-abdominal tumors with an unknown etiology with a prevalence of 1 per 20,000 children with 25% being diagnosed before the age of 10. They are often located in the smallbowel mesentery and up to a third of patients have multiple cysts. However, they are most commonly singular, are multiloculated, and rarely contain chylous fluid (2, 3). Chylous mesenteric cysts have an estimated incidence of 7.3% of all abdominal cysts (7). Often, these cysts can be detected during laparotomy or laparoscopy. Although often asymptomatic, 10% of patients with such cysts present as an acute abdomen (8). Both sexes are equally affected (9). It seem that this low frequency in children when compared to adults, is a consequence of late diagnosis (10). The physiopathology of chylous MC is still unknown. MC are usually developmental in origin but may arise as result of trauma, infection or malignancy (4). The prevalent hypothesis suggest that these lesions develop as consequence of benign lymphatic vessels proliferation which cause imbalance in inflow/outflow of chylous (4). Other hypothesis are failure of embryonic lymphatic vessels to join venous system (1) and non-fusion of mesenteric leaves results in accumulation of lymphatic fluid within this space (11). MC occurs more frequently in jejunum mesentery perhaps because in this bowel segment there is a greater lymph flow (6). Developmental cysts have further been classified as chylangioma (lymphangioma containing chylous) and chyle-filled lymphatic cysts (a dilated lymphatic developing due to an obstruction of the lymph flow) (5). It has been also reported that malignant transformation may occur in 3% of cases (9, 12). Clinical and radiological findings are usually non-specific. Simple radiological investigation such as plain abdominal x-ray and abdominal ultrasound may contribute to diagnosis but, often, only at surgery it is confirmed (1). At plain x-ray usual findings are air-fluid levels and shifting of intestinal loops due to mass effect. At sonography, a “fluid-fluid level” *sign* (13) or an “anaechoic cystic mass with diffuse, fine, non-dependent echoes” (14) have been described as suggestive of chylolymphatic cyst. Regardless of the type of lesion, an excision of the cyst (with or without involved bowel) is the treatment of choice for these lesions and such surgery is normally curative (6, 15). In our patient, the pathologist confirmed the MC as unilocular and with the absence of a lining epithelium and pseudo capsule. Based on this description we classified this MC as chyle filled lymphatic cyst originating from the non-fusion of the mesenteric leaves.

## Conclusion

Our reported case, can be considered one of the few MCC patients presenting as intestinal obstruction in early infancy. The need of differential diagnosis with lymphangioma or other abdominal cystic lesions and the curative role of MCC excision are important elements for the pediatric surgeon to consider MCC as a possible cause of intestinal obstruction. In fact, early detection of MCC may limit the need of intestinal resection of interested bowel (15). A prompt diagnosis, and an immediate and minimal surgical intervention guarantee an effective and definitive solution of this condition.

## Data Availability Statement

The raw data supporting the conclusions of this article will be made available by the authors, without undue reservation.

## Author Contributions

AS and FI conceived the study. VC, RC, FC, CM, and MN collected and analyzed the data. FB supervised the manuscript. All authors contributed to the article and approved the submitted version.

## Conflict of Interest

The authors declare that the research was conducted in the absence of any commercial or financial relationships that could be construed as a potential conflict of interest.
