# Supplementary material for: A Rare Case of Mesenteric Chylous Cyst in Infant: Case Report and Review of Literature
Source: Front Surg. 2021 Jun 14;8:666488. doi: 10.3389/fsurg.2021.666488 (PMC8236529; doi:10.3389/fsurg.2021.666488)
Supplement: Supplementary file 1 [file Data_Sheet_1.PDF]

A RARE CASE OF MESENTERIC CHYLOUS CYST IN INFANT

A 4-month old male infant was admitted for bloody stool, fever and bilious vomiting.

An exploratory laparoscopy is performed for diagnostic purposes.

A plain abdominal x-ray showed air fluid levels.

Abdominal US demonstrated only a large sub hepatic fluid collection.

Laparoscopy was converted in mini-laparotomy which confirmed the presence of MC in the first jejunal loop.

The jejunal loop wall was also affected by necrosis and inflammation so we decided to resect a limited segment of it (3 cm), followed by jejunal end to end anastomosis and appendectomy.

The specimens sent to pathologist confirmed that mesenteric leaves were interested by intense chronic inflammation without a lining epithelium.

The infant reached the full enteral feeding on day 3rd post-operative and discharged home in good condition at day 6th .
