# Supplementary figures and images for: A Rare Case of Mesenteric Chylous Cyst in Infant: Case Report and Review of Literature
Source: Front Surg. 2021 Jun 14;8:666488. doi: 10.3389/fsurg.2021.666488 (PMC8236529; doi:10.3389/fsurg.2021.666488)

## A RARE CASE OF MESENTERIC CHYLOUS CYST IN INFANT

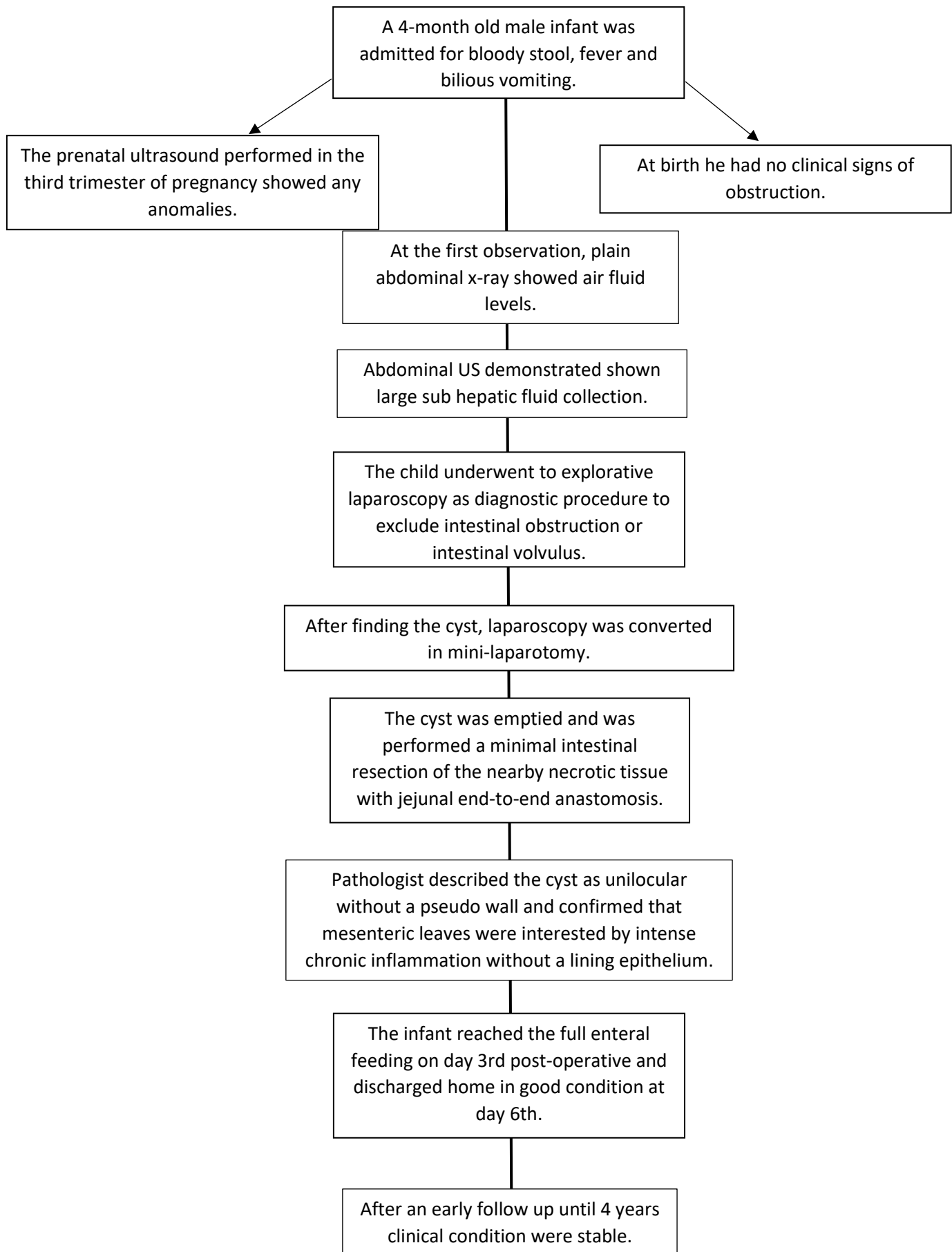

Supplement: Supplementary file 2 [file Data_Sheet_2.PDF]
